# Beyond arboviruses: A multicenter study to evaluate differential diagnosis of rash diseases and acute febrile illness cases in Rio de Janeiro, Brazil

**DOI:** 10.1371/journal.pone.0271758

**Published:** 2022-07-29

**Authors:** Arthur Daniel Rocha Alves, Jéssica Vasques Raposo, Rafaela Moraes Pereira de Sousa, Claudete Aparecida Araújo Cardoso, Pâmela Karla Simões de Freitas Costa, Julienne Martins Araújo, Sabrina Teresinha Alvim Barreiro, Clarisse da Silveira Bressan, Guilherme Amaral Calvet, Rogério Valls de Souza, Patrícia Brasil, Rita de Cássia Nasser Cubel Garcia, Marcelo Alves Pinto, Vanessa Salete de Paula, Luciane Almeida Amado

**Affiliations:** 1 Laboratório de Desenvolvimento Tecnológico em Virologia, Instituto Oswaldo cruz, Fundação Oswaldo Cruz, Rio de Janeiro, RJ, Brasil; 2 Laboratório de Virologia Molecular, Instituto Oswaldo cruz, Fundação Oswaldo Cruz, Rio de Janeiro, RJ, Brasil; 3 Departamento Materno Infantil, Faculdade de Medicina, Universidade Federal Fluminense, Niterói, RJ, Brasil; 4 Hospital Getúlio Vargas Filho, Secretaria Municipal de Saúde, Niterói, RJ, Brasil; 5 Laboratório de Pesquisa Clínica em Doenças Febris Agudas, Instituto Nacional de Infectologia Evandro Chagas, Rio de Janeiro, RJ, Brasil; 6 Departamento de Microbiologia e Parasitologia, Instituto Biomédico, Universidade Federal Fluminense, Niterói, RJ, Brasil; Universitas Syiah Kuala, INDONESIA

## Abstract

**Introduction:**

A wide variety of viruses can cause rash diseases (RDs) or acute febrile illness (AFIs) in children, adolescents and adults; however, approximately 19% of RD cases and 40% of AFI cases remain without a defined etiology. Parvovirus B19 (B19V) and herpesvirus infection can also cause RD and/or AFI, and in some risk groups, these infections can become persistent (or latent) and may require hospital treatment. Since these infections do not have mandatory reporting, they can be hidden by other diseases, such as those caused by arboviruses (*e*.*g*., dengue virus). In this context, the aim of this study was to pursue the differential laboratory diagnoses of B19V and herpesvirus infections in patients with RD and AFI, without a defined etiology, seen in hospitals and/or reference centers for infectious diseases in Rio de Janeiro.

**Methods:**

A total of 114 participants were enrolled in the study, including 54 children and 60 adults. B19V infection was assessed by real-time PCR (qPCR) and ELISA (anti-B19V IgM and IgG). EBV was assessed through qPCR, and betaherpesviruses (HCMV, HHV-6 and HHV-7) were assessed through multiplex qPCR. Sociodemographic and clinical data were obtained from the medical record data of these participants.

**Results:**

The median age of children with RD was 2 years (interquartile range (IQR): 5), and 55.6% were male. Among adults with AFI, the median age was 38 years (IQR: 21), and 56.7% were female. Regarding RD patients, viral prevalence (and load) were 5.5%(10^4^IU/mL), 3.4%(10^4^IU/mL), 5.5%(10^4^IU/mL) and 11.1%(10^5^IU/mL) for B19V, EBV, HCMV and HHV-6 infection, respectively, and in AFI patients they were 6.6%(10^5^IU/mL), 1.6%(10^3^IU/mL), 3.3%(10^4^IU/mL) for B19V, HCMV and HHV-6, respectively. HHV-7 was not detected in RD or AFI patients.

**Conclusion:**

These results suggest the importance of including B19V and herpesviruses in the differential laboratory diagnoses for patients with RD and AFI, not only for epidemiological purposes but also for the proper management of the patient.

## Introduction

A wide variety of viruses share the ability to cause rashes that can be localized or disseminated in skin and associated or not associated with exanthema, fever, malaise, pruritus and other systemic signs and symptoms [1]. So-called viral rash diseases (RDs) are extremely common in Brazil and constitute a serious public health problem, as they are usually the main cause of seeking medical care in hospitals and pediatric clinics. However, there are several difficulties in determining an accurate etiological diagnosis, especially if etiological identification occurs exclusively based on clinical examination [2–5].

The most common etiological agents for RD include measles virus, rubella virus, dengue virus, chickenpox virus, cytomegalovirus, Epstein Barr virus, human herpesvirus 6, human herpesvirus 7, enterovirus, human parvovirus B19, chikungunya virus and Zika virus [3, 4]. Since most of these infectious agents share many clinical manifestations, the etiological diagnosis of RD is of paramount importance, not only for guiding the treatment of choice but also for epidemiological purposes, such as controlling and eradicating infections.

Acute febrile illnesses (AFIs) may or may not be associated with the presence of skin rash. AFI is characterized by fever lasting less than seven days without identifying a bacterial source [6, 7]. Fever is a common reason for seeking health care in Latin America and is associated with substantial morbidity and mortality [8]. As a typical feature of many infectious and noninfectious diseases, the etiological diagnosis of fever represents a considerable challenge for health care professionals and surveillance systems, especially when confirmatory tests are not available at the place of care [9]. Environmental conditions, socioeconomic factors and availability of vaccines are some of the factors that influence the incidence and etiology of febrile infectious diseases in the region.

A recently published review on the epidemiology of AFI in Latin America showed that between 2007 and 2016, in the city of Rio de Janeiro, 40% of cases of AFI did not have a defined etiology, which highlights an important gap in current knowledge that should be addressed to improve the understanding of AFI in the region [10].

In the Brazilian epidemiological scenario, where several emerging and reemerging viruses cocirculate, such as the dengue, chikungunya and Zika viruses, together with the increasing trends of measles cases, demand the attention of public health agencies [11]. According to the current Brazilian epidemiological bulletin, the incidence of dengue and chikungunya varies from moderate to high depending on the state, with an overall incidence in the country of 726 cases per 100,000 inhabitants and 62 cases per 100,000 inhabitants, respectively. In addition, cases of Zika virus are still reported, reflecting an overall incidence of 5.1 cases per 100,000 inhabitants [12]. These data demonstrate the high investigation and incidence of arboviruses in Brazil that could hide other infections, as has been demonstrated by Di Paola and collaborators [13].

The investigation of RD and fever illness etiologies other than emerging pathogens is needed and would probably reveal a higher burden of neglected pathogens, such as Parvovirus B19 and herpesviruses (Cytomegalovirus, Epstein Barr, Human Herpesvirus 6, and Human Herpesvirus 7). These infections have been underestimated and underdiagnosed [13], and although they represent in most cases benign and self-limited diseases, in some cases, there is a risk of progressing to a more serious clinical course [14–16].

Parvovirus B19 usually causes a common childhood RD known as erythema infectiosum (EI), arthropathy in adults and aplastic crisis in patients with hemolytic diseases. In immunocompromised patients, B19V infection is not readily cleared, and its long persistence leads to chronic anemia. During pregnancy, adverse outcomes, such as severe fetal anemia and hydrops fetalis, can occur [17]. Approximately 19% of RD cases remain without a confirmatory diagnosis, and among cases with defined etiology, infection by human Parvovirus B19 is the main cause (33%), demonstrating the need to perform specific laboratory diagnosis [8].

In Brazil, the epidemiological scenario of B19V was recently reviewed [18], and we observed that the period of study was from 1987 to 2016 in specific states of the country and varied from 2% (Pará state in 1987) to 63.2% (São Paulo state in 2013–2014). In relation to herpesviruses, the seroprevalence of EBV, CMV, HHV-6 and HHV-7 has been evaluated in some studies with groups of patients with rash diseases of unknown etiology [19–23], and we observed that the period of study was from 1996 to 2011 in specific states of the country and varied from 2.0% (Pará state in 1997) to 3.1% (São Paulo state in 2003–2004) for EBV, 17.0% (São Paulo state in 2000–2004) to 63.8% (Pará state in 1997) for HHV-6, and 8.0% (São Paulo state in 1992–1993) to 51.4% (Pará state in 1996–2002) for HHV-7. Therefore, there have been no epidemiological efforts to present the real dynamics of this infection in Brazil in recent years. Previous studies have already indicated the relevance of implementing the differential diagnoses for the aforementioned viruses in cases of RD without a defined etiology.

Infections caused by herpesviruses are also often asymptomatic, benign, and self-limiting, and the ability to remain in latency in the host is an intrinsic characteristic of all members of the Herpesviridae family. In line with viral latency, greater severity and reactivations of herpetic infections are expected in immunosuppressed patients in specific age groups, such as childhood and senility, as well as during pregnancy. In severe and untreated cases, herpesvirus infections can lead to death. Even though most of these infections have a benign and self-limiting course, for certain age groups, pregnant and immunocompromised patients, they could represent an important risk, as they can progress to severe diseases that may require specific treatment and emergency hospitalization, burdening the public health service [24]. Therefore, investigation of B19V and herpesviruses as a differential diagnosis of RD and fever illness is of great importance to ensure adequate treatment of the patient and to improve the surveillance systems of these infections, which include implementation of diagnostic tools and establishment of measures for disease control.

Thus, the aim of this study was to determine the differential diagnosis of parvovirus (B19V) and herpesvirus (CMV, EBV, HHV-6 and 7) among individuals with RD and AFI without a defined etiology to identify their prevalence and clinical characteristics, aiming to contribute to understanding the RD and AFI fever epidemiology seen in hospitals and/or reference centers for infectious diseases in Rio de Janeiro, Brazil.

## Materials and methods

### Study population and ethical aspects

From December 2018 to October 2019, children and adolescents aged between 0 and 18 years who attended Hospital Getúlio Vargas Filho (HGVF) and Hospital Universitário Antônio Pedro (HUAP) (Niterói, RJ) with RD were invited to participate in this study. Entry criteria of RD disease included a macular, maculopapular, papular, urticarial, or vesicular diffuse cutaneous eruption commonly accompanied by prodromal systemic clinical manifestations such as fever and malaise, in addition to respiratory or gastrointestinal signals and symptoms. All the participants were less than 10 days from symptom onset.

From May 2018 to October 2019, adult patients with AFI treated at the Instituto Nacional de Infectologia Evandro Chagas (INI) (Rio de Janeiro, RJ) were invited to participate in this study. Inclusion criteria were men and women aged ≥18 years who had developed acute fever with or without a rash and no evident focus of infection within the previous seven days. We defined fever as an axillary temperature of ≥ 37.5°C. During the study period, 330 patients presenting with RD and/or AFI were attended to at HGVF, HUAP and INI, and out of these, 216 (65.5%) had at least one type of arbovirus detectable by molecular methods (dengue virus, yellow fever virus, Zika virus and/or chikungunya virus). Chikungunya was the arbovirus responsible for most (88%) of the RD or AFI cases, while 34.7% of the RD and AFI cases had no defined etiology. Only patients without a defined etiology for RD or AFI were included in this study.

All patients with RD or AFI who had acute B19V infection diagnosed in the present work were followed to evaluate the outcomes of the infection. At the time of the interview, the signs and symptoms of the patients were reported. To maintain the same routine of follow-up and return of patients in each hospital, these patients were monitored as follows: patients attended to at HGVF and HUAP were followed for 6 months, with consecutive serum samples collected at 1 month, 3 months and 6 months after the initial collection; patients attended at INI were followed for 2 months with consecutive serum samples collected at 1 week, 1 month and 2 months after the initial collection. One AFI patient attended at INI did not return for subsequent sample collections for the present study.

The study protocol was approved by the Ethics Committee of Oswaldo Cruz Institute (Protocols #2.498.339 and # 3.627.364) as well as by all partnering health units. Parents or guardians of patients under 18 years of age provided written informed consent. Patients aged 18 years and over provided written informed consent; patients aged 7–17 years provided written assent to participate.

### Molecular and serologic tests for B19V

B19V infection was investigated in the serum samples collected at the day of the medical appointment through serological and molecular assays, after the consent of the patient.

For molecular detection and quantification of B19V, DNA was extracted from serum samples using a QIAamp DNA Blood Mini Kit (Qiagen, Hilden, Germany). Real-time PCR was carried out using the TaqMan system (Applied Biosystems 7500 Real-Time PCR System, Applied Biosystems, USA), as described by Alves and collaborators [25].

To determine whether B19V DNA-positive patients had acute or persistent infection, all serum samples positive for B19V DNA were investigated for the presence of anti-B19V IgM and IgG antibodies through an enzyme-linked immunosorbent assay (Virion Serion, Brazil) according to the manufacturer’s instructions.

For B19V genotyping, nested PCR [26] using the primer pairs VP1/VP1R (nt 3502–3883, GenBank: NC_000883.2) and VP2/VP2R (nt 3541–3867, GenBank: NC_000883.2) was performed to amplify the 326 bp fragment of the VP1/VP2 capsid gene. The nested PCR products were purified using QIAquick gel extraction (Qiagen, Germany) and then sequenced directly using the BigDye terminator v. 1.1 cycle sequencing kit (Applied Biosystems, USA) and an ABI Prism^®^ 3730 DNA analyzer (Applied Biosystems, USA). Both strands of each amplicon were sequenced.

For phylogenetic analysis, sequences were aligned using the BioEdit Sequence Alignment Editor v. 7.2.5. A maximum likelihood tree was constructed based on the Tamura-Nei+G+I model and inferred using a bootstrap consensus tree from 1000 replicates, available in MEGA v. X software [27]. The B19V genotype was determined by including reference sequences of genotypes 1A, 1B, 2, 3A and 3B available in GenBank. To root the tree, a sequence from Human Bocavirus available in GenBank was used.

### Molecular tests for herpesviruses

Herpesvirus infection was also investigated in the serum samples after DNA extraction using a QIAamp DNA Blood Mini Kit (Qiagen, Hilden, Germany). EBV was detected and quantified through real-time PCR (qPCR) [28], and betaherpesviruses (HCMV, HHV-6 and HHV-7) were detected through multiplex qPCR [29]. For amplification of HCMV, HHV-6 and HHV-7, specific probes and oligonucleotides were used for the UL54 region (referring to DNA synthesis, 130 bp), U56 region (referring to the viral capsid, 150 bp) and U37 region (referring to the tegument of the virus, 312 bp), respectively. The detection and quantification of EBV was performed by qPCR monoplex, using oligonucleotides and a probe for the target region EBNA-1, referring to the nuclear antigen EB (100 bp). All qPCR (monoplex and multiplex) procedures were performed using the TaqMan system (AgPath-ID PCR, Life Technologies, Carlsbad, USA).

### Statistical analysis

Statistical analysis was conducted using R studio (version 1.2.1335, Boston, USA). Continuous variables were expressed as the mean (or median, when applicable) and were compared with the Mann–Whitney U test. Categorical variables are expressed as numbers (%), and the Chi-square test or Fisher’s exact test was used. The odds ratio (OR) for virus detection in RD and/or AFI patients was analyzed with univariate logistic regression. All P values were two-sided, and those < 0.05 were considered statistically significant.

## Results

### Characterization of the studied population

During the period of this study, 54 children with RD with undefined etiology were included. Their median age was 6 years with an interquartile range (IQR) of 5 years, and 55.6% were male (Table 1).

**Table 1:** Demographical findings among children and adolescents with rash diseases (RD).

Parameter	Parvovirus B19		Cytomegalovirus		Epstein-Barr		Herpesvirus 6		Total (n = 54)
	B19V+ (n = 3)	P-value^a^; OR (95% CI)	HCMV+ (n = 3)	P-value^a^; OR (95% CI)	EBV+ (n = 2)	P-value^a^; OR (95% CI)	HHV-6+ (n = 6)	P-value^a^; OR (95% CI)	
Gender									
*Male n(%)*	2 (66.7)	0.69; 1.64 (0.14–19.28)	2 (66.7)	0.69; 1.64 (0.14–19.28)	2 (100)	0.35; 4.29 (0.19–93.90)	3 (50)	0.77; 0.77 (0.14–4.25)	30 (55.6)
*Female n(%)*	1 (33.3)		1 (33.3)		0 (0)		3 (50)		24 (44.4)
Age group (years)									
≤ *2 n(%)*	1 (33.3)	0.44; 0.38 (0.03–4.46)	2 (66.7)	0.69; 1.64 (0.14–19.28)	1 (50)	0.87; 0.79 (0.04–13.37)	6 (100)	0.08; 13.0 (0.69–243.58)	30 (55.6)
*> 2 n(%)*	2 (66.7)		1 (33.3)		1 (50)		0 (0)		24 (44.4)
*Median age; range*	6y; 9m-7y	0.73; NA	1y; 9m-7y	0.82; NA	4y; 2y-6y	0.39; NA	10m; 6m-1y	0.49; NA	2y; 6m-15y
Race / ethnicity									
*White n(%)*	1 (33.3)	0.51; 0.44 (0.04–5.22)	1 (33.3)	0.51; 0.44 (0.04–5.22)	2 (100)	0.31; 5.0 (0.23–109.21)	4 (66.7)	0.47; 1.91 (0.31–11.49)	28 (52.0)
*Black n(%)*	2 (66.7)		2 (66.7)		0 (0)		2 (33.3)		24 (44.4)
*Asian n(%)*	0 (0)		0 (0)		0 (0)		0 (0)		1 (1.8)
Weight (Kg) Mean±SD	13.3±11.3	0.52; NA	9.4±7.7	0.64; NA	12.2±8.5	0.79; NA	7.8±3.5	0.15; NA	17.2±10.5
Height (cm) Mean±SD	111.5±16.3	0.64; NA	64.0±46.1	0.07; NA	108.5±14.8	0.75; NA	73.5±5.1	**0.02; NA**	101.9±28.3

n: number; B19V: Parvovirus B19; EBV: Epstein-Barr Virus; HCMV: Cytomegalovirus; HHV-6: Human Herpesvirus 6; HHV-7: Human Herpesvirus 7; OR: *odds-ratio*; 95% CI: 95%Confidence interval; m: months; y: years; NA: non-appicable; SD: standard deviation

^a^ P-value referred to the Pearson *X*^2^ test.

Regarding individuals with acute febrile illnesses, 60 adults were included in the study. The median age was 38 years, the IQR was 21 years, and 56.7% were female (Table 2).

**Table 2:** Demographical findings among adults with acute febrile illness (AFI).

Parameter	Parvovirus B19		Cytomegalovirus		Herpesvirus 6		Total (n = 60)
	B19V+ (n = 4)	P-value^a^; OR (95% CI)	HCMV+ (n = 1)	P-value^a^; OR (95% CI)	HHV-6+ (n = 2)	P-value^a^; OR (95% CI)	
Gender							
*Male n(%)*	2 (50)	0.78; 1.33 (0.1–19.1)	0 (0)	0.60; 0.42 (0.01–10)	0 (0)	0.37; 0.24 (0.01–5.3)	26 (43.3)
*Female n(%)*	2 (50)		1 (100)		2 (100)		34 (56.7)
Age group (years)							
≤ *38 n(%)*	2 (50)	0.94; 0.93 (0.1–7.1)	1 (100)	2.91; 0.51 (0.1–74.1)	2 (100)	0.31; 5.00 (0.2–108)	31 (51.7)
*>38 n(%)*	2 (50)		0 (0)		0 (0)		29 (48.3)
*Median age; range*	40y; 22y-54y	0.97; NA	34y	NA	33y; 29y-68y	0.53; NA	38y; 18y-68y
Race / ethnicity							
*White n(%)*	1 (25)	0.11; 0.14 (0.01–1.5)	1 (100)	0.73; 1.76 (0.06–47)	2 (100)	0.47; 3.11 (0.13–71)	20 (33.3)
*Black n(%)*	3 (75)		0 (0)		0 (0)		11 (18.3)
*Unknown n(%)*	0 (0)		0 (0)		0 (0)		29 (48.4)

n: number; B19V: Parvovirus B19; EBV: Epstein-Barr Virus; HCMV: Cytomegalovirus; HHV-6: Human Herpesvirus 6; HHV-7: Human Herpesvirus 7; OR: *odds-ratio*; 95% CI: 95% Confidence interval; m: months; y: years; NA: non-appicable; SD: standard deviation

^a^ P-value referred to the Pearson *X*^2^ test.

### Molecular and serological B19V markers

B19V DNA was detected in three RD patients (5.5%; 3/54), with a mean (± standard deviation) viral load of 4.0x10^4^ (± 2.9x10^4^) IU/mL (Table 3). B19V DNA was also detected in four AFI patients (6.6%; 4/60), with a mean (± standard deviation) viral load of 3.0x10^5^ (± 1.3x10^4^) IU/mL (Table 3).

**Table 3:** Molecular Parvovirus B19 and Herpesviruses markers among rash diseases (RD) and acute febrile illness (AFI) patients attending in three different Hospitals in Rio de Janeiro during 2018–2019.

Etiological agent	RD (n = 54)		AFI (n = 60)		Total (n = 114)
	n (%)	Viral load (IU/mL; Mean ± SD)	n (%)	Viral load (IU/mL; Mean ± SD)	n (%)
B19V	3 (5.5)	4.0x10^4^ ± 2.9x10^4^	4 (6.6)	3.0x10^5^ ± 1.3x10^4^	7 (6.1)
EBV	2 (3.4)	7.1x10^4^ ± 4.2x10^1^	0 (0)	-	2 (1.8)
HCMV	3 (5.5)	3.9x10^4^ ± 2.2x10^4^	1 (1.6)	2.4x10^3^ ^a^	4 (3.5)
HHV-6	6 (11.1)	6.4x10^5^ ± 3.9x10^5^	2 (3.3)	3.1x10^4^ ± 4.2x10^4^	8 (7.0)
HHV-7	0 (0)	-	0 (0)	-	0 (0)
Total	14 (25.9)	-	7 (11.7)	-	21 (18.4)

B19V: Parvovirus B19; EBV: Epstein-Barr Virus; HCMV: Cytomegalovirus; HHV-6: Human Herpesvirus 6; HHV-7: Human Herpesvirus 7; IU/mL: International units per mililiter; RD: Rash diseases; AFI: Acute febrile illness; n: number; SD: standard deviation

^a^ As this infection was positive only in one patient, there is no mean ± SD, but only the viral load of this patient.

**Fig 1:**
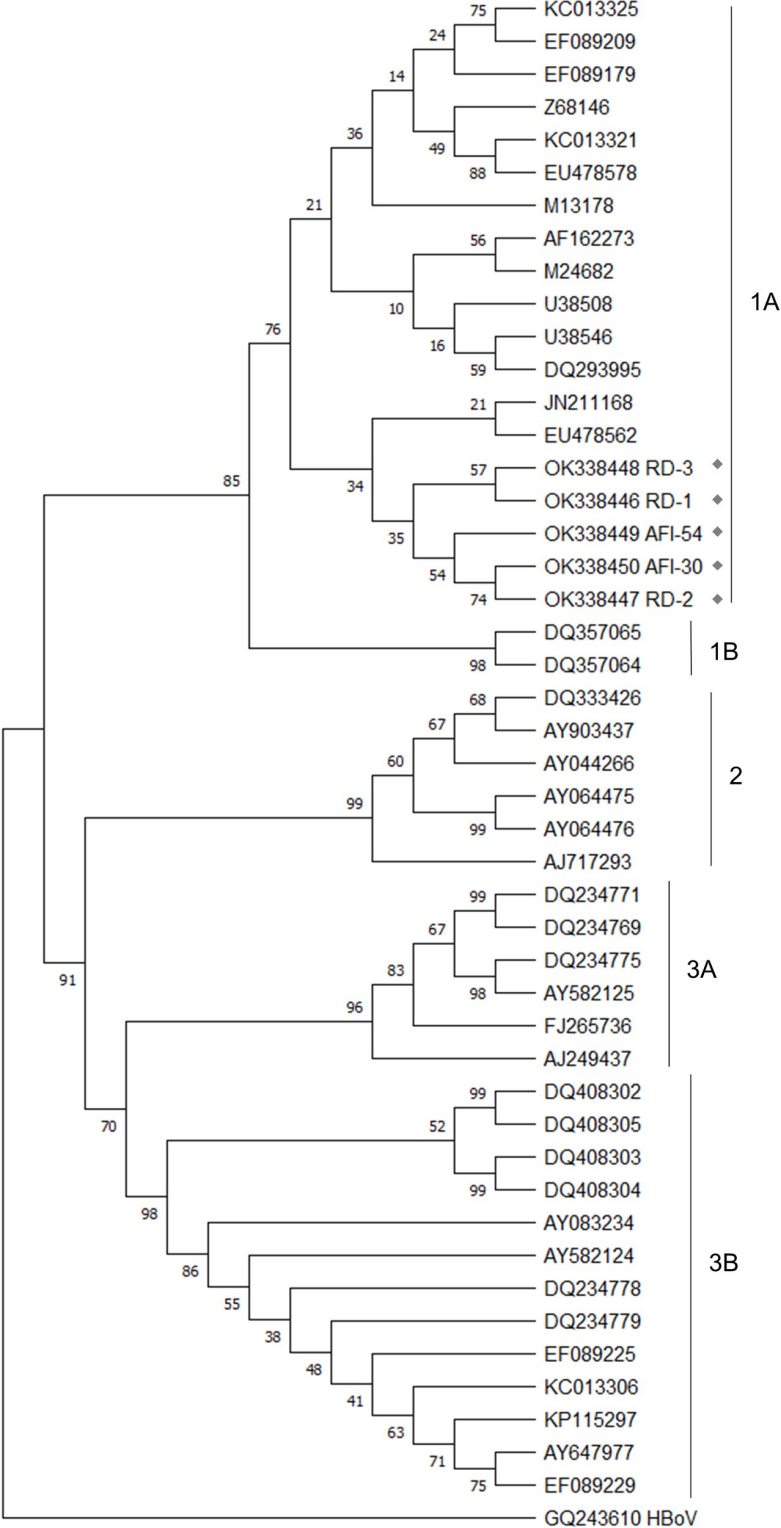
Evolutionary history of B19V genotypes, detected in patients with RD and AFI from Rio de Janeiro, Brazil between 2018 and 2019, inferred by using the Maximum Likelihood method and Tamura-Nei+G+I model. All analysis were conducted in Mega X. The samples of this study were marked by gray diamonds and named by RD and AFI prefix-numbers.

B19V isolated from RD (n = 3) and AFI (n = 2) patients were classified as genotype 1A (Fig 1). The nucleotide sequences obtained during this study were deposited in the GenBank database under accession numbers OK338446 to OK338450.

Overall, 6.1% (7/114) of the patients had B19V infection (Table 3). Anti-B19V IgM and IgG were investigated among these B19V DNA-positive RD and AFI patients (n = 7) to evaluate acute or persistent infection. Anti-B19V IgG was found in 100% (7/7) of patients, and anti-B19V IgM was detected in 71.4% (5/7) of patients. Among anti-B19V IgM-positive patients, 66.7% (2/3) were RD patients, and 75.0% (3/4) were AFI patients.

During the follow-up, all consecutive samples (from RD collected at 1 month, 3 months and 6 months and from AFI collected at 1 week, 1 month and 2 months) were negative for B19V DNA (Table 4).

**Table 4:** Follow-up of molecular and serological markers of Parvovirus B19 among rash diseases (RD) and acute febrile illness (AFI) patients attending in three different Hospitals in Rio de Janeiro during 2018–2019.

Rash Diseases																
Patient	Age (years)	Gender	Hb (g/dL)^a^	Onset of symptoms	0			2 months			3 months			6 months		
					DNA	IgM	IgG	DNA	IgM	IgG	DNA	IgM	IgG	DNA	IgM	IgG
#1	7	M	**9,6**	1	+	+	+	-	-	+	-	-	+	-	-	+
#2	6	F	**9,5**	2	+	+	+	-	+	+	-	-	+	-	-	+
#3	1	M	**9,5**	1	+	+	+	-	+	+	-	-	+	-	-	+
Acute Febrile Illness																
Patient ^b^	Age (years)	Gender	Hb (g/dL)^a^	Onset of symptoms	0			1 week			1 month			2 months		
					DNA	IgM	IgG	DNA	IgM	IgG	DNA	IgM	IgG	DNA	IgM	IgG
#25	22	F	15,1	6	+	-	+	-	-	+	-	-	+	-	-	+
#29	50	M	**12,6**	4	+	+	+	-	+	+	-	+	+	-	+	+
#30	54	M	**12,1**	4	+	+	+	-	+	+	-	+	+	-	-	+

B19V: Parvovirus B19; Hb: Hemoglobin; IU/mL: International units per mililiter; IgM: Immunoglobulin M; IgG: Immunoglobulin G; -: negative; +: positive; RD: Rash diseases; AFI: Acute febrile illness.

^a^ In bold are patients considered to have anemia;

^b^ One female AFI patient (#54) positive for B19V was not included in this table, because she did not return for subsequent sample collections of this study

### Molecular EBV, HCMV, HHV-6, HHV-7 markers

EBV DNA was detected in two RD patients (3.4%; 2/54), with a mean (± standard deviation) viral load of 7.1x10^4^ ± 4.2x10^1^ copies/mL, and was not detected in AFI patients (Table 3). HCMV DNA was detected in three RD patients (5.5%; 3/54), with a mean (± standard deviation) viral load of 3.9x10^4^ ± 2.2x10^4^ copies/mL, and in one AFI patient (1.6%; 1/60), with a viral load of 2.4x10^3^ copies/mL (Table 3). HHV-6 DNA was detected in six RD patients (11.1%; 6/54), with a mean (± standard deviation) viral load of 6.4x10^5^ ± 3.9x10^5^ copies/mL, and in two AFI patients (3.3%; 2/60), with a mean (± standard deviation) viral load of 3.1x10^4^ ± 4.2x10^4^ copies/mL (Table 3). HHV-7 DNA was not detected in RD or AFI patients (Table 3).

A total of 11 RD patients (20.4%) and three AFI patients (5.0%) were infected by a herpesvirus; in total, 14/114 (12.3%) of the patients in this study had a herpesvirus infection (Table 3).

### Clinical manifestations and biochemical analysis among RD patients

According to Table 5, six of the 54 RD patients had anemia (11.1%), with a mean hemoglobin level of 11.4 ± 1.2 g/dL. All B19V DNA-positive patients (3/3) had anemia (OR = 73.0, p = 0.007, CI = 3.09–1720.1), headache (OR = 31.32, p = 0.02, CI = 1.49–658.3), and head as a local rash (OR = 24.65, p = 0.03, CI = 1.18–512.6). In the blood count of these patients, the hemoglobin level (p = 0.0014), hematocrit (p = 0.04) and neutrophils (p = 0.04) were significantly lower than those of the RD-B19V-negative patients, while the lymphocyte count (p = 0.03) and C-reactive protein level (p = 0.002) were significantly higher than those of the RD-B19V-negative patients (Table 5).

**Table 5:** Clinical and laboratorial findings among children and adolescents with rash diseases (RD).

Parameter	Parvovirus B19		Cytomegalovirus		Epstein-Barr		Herpesvirus 6		Total (n = 54)
	B19V+(n = 3)	P-value^a^; OR (95% CI)	HCMV+(n = 3)	P-value^a^; OR (95% CI)	EBV+(n = 2)	P-value^a^; OR (95% CI)	HHV-6+ (n = 6)	P-value^a^; OR (95% CI)	
Fever n(%)	2 (66.7)	0.70; 0.62 (0.05–7.39)	3 (100)	0.58; 2.45 (0.11–50.67)	0 (0)	0.06; 0.05 (0.002–1.23)	6 (100)	0.18; 7.14 (0.38–133.60)	41 (75.9)
Headache n(%)	3 (100)	**0.02; 31.32 (1.49–658.3)**	2 (66.7)	0.09; 8.2 (0.67–99.70)	0 (0)	0.78; 0.64 (0.02–14.41)	0 (0)	0.42; 0.29 (0.01–5.66)	12 (22.2)
Myalgia n(%)	1 (33.3)	0.44; 2.69 (0.22–33.28)	1 (33.3)	0.44; 2.69 (0.22–33.28)	0 (0)	0.96; 0.92 (0.04–20.66)	1 (16.7)	0.98; 1.02 (0.10–9.98)	9 (16.7)
Arthralgia n(%)	1 (33.3)	0.12; 8.00 (0.55–115.4)	0 (0)	0.24; 11.22 (0.19–635.7)	0 (0)	0.63; 2.15 (0.08–52.16)	0 (0)	0.85; 0.76 (0.03–15.83)	4 (7.4)
Nausea and vomiting n(%)	1 (33.3)	0.64; 0.56 (0.04–6.60)	2 (66.7)	0.47; 2.43 (0.21–28.58)	0 (0)	0.32; 0.21 (0.009–4.71)	1 (16.7)	0.16; 0.21 (0.02–1.91)	25 (46.3)
Diarrhea n(%)	1 (33.3)	0.76; 1.46 (0.12–17.48)	1 (33.3)	0.76; 1.46 (0.12–17.48)	0 (0)	0.68; 0.53 (0.02–11.73)	2 (33.3)	0.62; 1.58 (0.25–9.74)	14 (25.9)
Tachycardia n(%)	1 (33.3)	0.18; 5.87 (0.43–79.78)	1 (33.3)	0.18; 5.87 (0.43–79.78)	0 (0)	0.73; 1.72 (0.07–40.80)	0 (0)	0.74; 0.60 (0.03–12.34)	5 (9.3)
Rash									
*Time (days;* Mean±SD*)*	1.3±0.6	0.36; NA	3.3±2.5	0.35; NA	2.5±2.1	0.94; NA	2.1±1.7	0.63; NA	2.6±2.5
*Local—head n(%)*	3 (100)	**0.03; 24.65 (1.18–512.6)**	2 (66.7)	0.30; 3.66 (0.31–43.27)	0 (0)	0.16; 7.66 (0.42–139.26)	3 (50)	0.49; 1.82 (0.33–10.05)	20 (37.0)
*upper body n(%)*	0 (0)		1 (33.3)		0 (0)		3 (50)		18 (33.3)
*upper limbs n(%)*	0 (0)		0 (0)		0 (0)		0 (0)		3 (5.6)
*lower limbs n(%)*	0 (0)		0 (0)		1 (50)		0 (0)		7 (12.9)
*diffuse n(%)*	0 (0)		0 (0)		1 (50)		0 (0)		6 (11.2)
*Scattering n(%)*	3 (100)	0.84; 1.37 (0.06–28.97)	3 (100)	0.84; 1.37 (0.06–28.97)	1 (50)	0.20; 0.15 (0.08–2.78)	6 (100)	0.30; 4.65 (0.24–87.89)	46 (85.2)
Anemia									
*Yes n(%)*	3 (100)	**0.007; 73.0 (3.09–1720.1)**	2 (66.7)	**0.03; 17.5 (1.28–238.91)**	0 (0)	0.97; 1.06 (0.04–24.77)	3 (50)	**0.01; 11.0 (1.5–80.5)**	6 (11.1)
*No n(%)*	0 (0)		1 (33.3)		2 (100)		3 (50)		36 (66.7)
MMR Vaccine									
*Yes n(%)*	3 (100)	0.91; 1.18 (0.05–25.23)	3 (100)	0.91; 1.18 (0.05–25.23)	2 (100)	0.90; 0.82 (0.03–18.91)	5 (83.3)	0.77; 0.71 (0.07–7.20)	47 (87.0)
*No n(%)*	0 (0)		0 (0)		0 (0)		1 (16.7)		7 (13.0)
Blood count (Mean±SD)									
*Hemoglobin level (g/dL)*	9.5±0.6	**0.0014; NA**	11.1±2.6	0.53; NA	12.8±3.7	0.21; NA	11.2±1.0	0.56; NA	11.4±1.2
*Hematocrit (%)*	31.1±1.3	**0.04; NA**	33.8±4.7	0.78; NA	20.5±20.2	**0.0001; NA**	33.7±4.1	0.37; NA	34.9±3.4
*Platelets (miles/mm*³*)*	251±174	0.38; NA	301±194	**0.01; NA**	384±208	0.64; NA	298±69	0.59; NA	331±163
*Leucocyte (miles/mm*³*)*	5500±3700	0.19; NA	6553±4110	**0.005; NA**	13400±5232	0.13; NA	6233±1699	0.12; NA	8786±4433
*Neutrophils (miles/mm*³*)*	28±8	**0.04; NA**	32±10	**0.0002; NA**	44±1.4	0.68; NA	41±12	0.25; NA	49±16
*Lymphocyte (miles/mm*³*)*	62±10	**0.03; NA**	62±10	0.38; NA	44±8.5	0.79; NA	51±11	0.15; NA	40±19
*C-reactive protein (mg/L)*	67.4±24.9	**0.002; NA**	61.4±26.7	0.15; NA	25.5±0.7	0.95; NA	1.2±0.4	**0.01; NA**	26.5±27.1

n: number; B19V: Parvovirus B19; EBV: Epstein-Barr Virus; HCMV: Cytomegalovirus; HHV-6: Human Herpesvirus 6; HHV-7: Human Herpesvirus 7; OR: *odds-ratio*; 95% CI: 95%Confidence interval; m: months; y: years; NA: non-appicable; SD: standard deviation

^a^ P-value referred to the Pearson *X*^2^ test.

Two of three HCMV DNA-positive patients (66.7%) had anemia (OR = 17.5, p = 0.03, CI = 1.28–238.91). In the blood count of HCMV DNA-positive patients, the platelets (p = 0.01), leukocytes (p = 0.005) and neutrophils (p = 0.0002) were significantly lower than those in the RD-HCMV-negative patients (Table 5).

In the blood count of EBV DNA-positive patients, the hematocrit (p = 0.0001) was significantly lower than that in the RD-EBV-negative patients (Table 5).

Among the HHV-6-positive patients, three (50%) had anemia (OR = 11.0, CI = 1.5–80.5, p = 0.01). The height (p = 0.02) and C-reactive protein (p = 0.01) of these patients were significantly lower than those of RD-HHV-6-negative patients (Table 5).

A significant relationship was not observed between the presence of B19V or herpesvirus DNA and age, gender, race (or ethnicity), number of people living at home, fever, myalgia, arthralgia, nausea and vomiting, diarrhea, tachycardia, or MMR vaccine (p>0.05; Table 5).

### Clinical manifestations and biochemical analysis among AFI patients

According to Table 6, among the individuals with AFI infected with B19V, only the presence of anemia was considered statistically significant (OR = 10.6, CI = 1.1–112, p = 0.04). A significant relationship between the presence of B19V or herpesvirus DNA and age, gender, race (or ethnicity), fever, headache, retroorbital pain, myalgia, arthralgia, nausea and vomiting, rash, and blood count was not observed (p>0.05; Table 6).

**Table 6:** Clinical and laboratorial findings among adults with acute febrile illness (AFI).

Parameter	Parvovirus B19		Cytomegalovirus		Herpesvirus 6		Total (n = 60)
	B19V+(n = 4)	P-value^a^; OR (95% CI)	HCMV+(n = 1)	P-value^a^; OR (95% CI)	HHV-6+ (n = 2)	P-value^a^; OR (95% CI)	
Fever n(%)	3 (75)	0.91; 0.88 (0.08–9.2)	0 (0)	0.14; 0.08 (0.03–2.2)	2 (100)	0.80; 1.48 (0.06–32)	47 (78.3)
Headache n(%)	2 (50)	0.72; 0.69 (0.09–5.3)	1 (100)	0.63; 2.21 (0.08–56)	0 (0)	0.19; 0.13 (0.06–2.8)	35 (58.3)
Retroorbital pain n(%)	0 (0)	0.59; 0.44 (0.02–8.7)	1 (100)	0.11; 14.1 (0.5–371)	1 (50)	0.28; 4.8 (0.27–83)	11 (18.3)
Myalgia n(%)	1 (25)	0.49; 0.44 (0.04–4.5)	0 (0)	0.63; 0.45 (0.01–11)	0 (0)	0.39; 0.26 (0.01–5.7)	25 (41.7)
Arthralgia n(%)	2 (50)	0.62; 0.60 (0.07–4.5)	0 (0)	0.33; 0.20 (0.07–5.1)	1 (50)	0.73; 0.61 (0.03–10)	37 (61.7)
Nausea and vomiting n(%)	1 (25)	0.93; 1.10 (0.10–11)	0 (0)	0.97; 1.04 (0.04–27)	0 (0)	0.75; 0.61 (0.02–13)	14 (23.3)
Rash n (%)	2 (50)	0.94; 0.93 (0.12–7.1)	1 (100)	0.51; 2.90 (0.11–74)	1 (50)	0.96; 0.93 (0.05–15)	31 (51.7)
*Time (days;* Mean±SD*)*	4.5±2.1	0.95; NA	1	NA	3	NA	4.2±3.7
*Local n (%) head*	0 (0)	0.25; 6.11 (0.26–138)	0 (0)	0.46; 3.41 (0.12–90)	0 (0)	0.46; 3.41 (0.12–90)	4 (6.7)
*upper body*	0 (0)		0 (0)		0 (0)		3 (5)
*upper limbs*	0 (0)		0 (0)		0 (0)		3 (5)
*lower limbs*	0 (0)		0 (0)		0 (0)		6 (10)
*diffuse*	2 (50)		1 (100)		1 (50)		15 (25)
Anemia							
*Yes n(%)*	3 (75)	**0.04; 10.6 (1.1–112)**	0 (0)	0.95; 0.90 (0.03–23)	0 (0)	0.68; 0.53 (0.02–11)	14 (23.3)
*No n(%)*	1 (25)		1 (100)		2 (100)		40 (66.7)
*Unknown n(%)*	0 (0)		0 (0)		0 (0)		6 (10)
Blood count (Mean±SD)							
*Hemoglobin level (g/dL)*	12.9±1.4	0.57; NA	13.3	NA	12.8±0.6	0.56; NA	13.4±1.5
*Hematocrit (%)*	36.9±3.6	0.30; NA	39.2	NA	37.5±0.8	0.60; NA	38.9±4.1
*Platelets (miles/mm*³*)*	313±59.8	0.06; NA	294	NA	262±56.6	0.72; NA	241±81.9
*Leucocyte (miles/mm*³*)*	5975±862	0.38; NA	8560	NA	8530±678	0.63; NA	7401±3394
*Neutrophils (miles/mm*³*)*	57.5±21.5	0.74; NA	65	NA	50.5±12	0.37; NA	59.9±14.9
*Lymphocyte (miles/mm*³*)*	29±16.7	0.91; NA	24	NA	37±11.3	0.47; NA	29.8±14.3
*C-reactive protein (mg/L)*	0.8±0.7	0.32; NA	0.89	NA	4.7±6.3	0.43; NA	2.7±3.7

n: number; B19V: Parvovirus B19; EBV: Epstein-Barr Virus; HCMV: Cytomegalovirus; HHV-6: Human Herpesvirus 6; HHV-7: Human Herpesvirus 7; OR: *odds-ratio*; 95% CI: 95% Confidence interval; m: months; y: years; NA: non-appicable; SD: standard deviation

^a^ P-value referred to the Pearson *X*^2^ test.

## Discussion

In this study, through the differential diagnosis of parvovirus (B19V) and herpesvirus (CMV, EBV, HHV-6 and 7), we identified the etiology of RD and AFI diseases whose diagnosis was not possible using only the clinical diagnosis.

Of the patients enrolled in the study, 14 received a clinical diagnosis compatible with rash diseases (caused by infectious agents such as scarlet fever, herpesvirus 6, Zika, chikungunya, coxsackie, dengue, measles, and chickenpox, or due to other causes, such as Kawasaki, pharmacodermia and pancytopenia). Of these patients, only one case of herpesvirus 6 infection was confirmed by laboratory diagnosis. However, two patients were diagnosed by epidemiological links and clinical presentation with infection by Chikungunya and measles viruses, respectively, but molecular diagnosis revealed the presence of Epstein–Barr and HHV-6 viruses. These findings demonstrate the importance of the differential laboratory diagnosis in patients with RD. It is also worth noting that the clinicians who attended these cases did not observe a clinical compatibility with cytomegalovirus and parvovirus B19.

The assessment of B19V incidence among individuals with RD and/or AFI varies widely in the literature according to geographical location and time. Furthermore, these studies differed largely in terms of study design and laboratory approach. In this study, an overall prevalence of B19V infection of 6.1% (7/114) was observed. A study from Spain reported laboratory-confirmed B19V infection in 33.42% of the 368 RD cases studied, showing that B19V was the main etiological agent of RD [30]. Epidemiological data on B19V infection among adults are scarce, but it has been demonstrated that 40% of adults with AFI have an undefined etiology [10]. Other studies detected 7.2% (33/456) of B19V in Kerala, India [31], 35% (322/906) between 2005 and 2008 in Belarus [32], and 4.4% of B19V using metagenomic NGS in Tororo, Uganda [33].

In Brazil, a recently published review [18] showed that the incidence of B19V in RD cases varied from 1.5% (6/401) in 1997 in Pará state (north region) [20] to 63.2% (115/182) between 2013 and 2014 in São Paulo state (southeastern region) [13]. In Rio de Janeiro state (southeastern region), the B19V incidence varied from 2.3% in 1992–1994 to 31.5% in 1994–1999 [34–37].

An important characteristic in Parvovirus B19 infection in Brazil is its cyclical pattern of occurrence between 4 and 5 years, including years with high infection rates followed by periods with low incidences, as happened in 1988–1989, 1995–1996, 1999–2000, 2004–2005, 2009–2010, and 2013–2014 [18, 38, 39]. After this period, there was no observational study that demonstrated epidemic years of B19V infection.

As all B19V-positive patients became DNA negative during the follow-up study, it is possible to assume that all patients had acute self-limiting infection. In one patient (AFI #25), there was no anti-B19V IgM at any point during the follow-up. We can hypothesize that the infection in this patient was in the past, as happens in other cases of B19V infection [40, 41]; alternatively, a lack of detection of IgM in this case could be due to immunocomplexes that may exist in acute B19V infection, resulting in negative anti-B19V IgM testing [42].

This study simultaneously evaluated all betaherpesviruses and the Epstein–Barr virus in RD and AFI populations. To date, no other study has evaluated these herpesviruses in the same population. Although it is difficult to express the clinical features of exanthem subitum quantitatively, during Days 3 and 4 of the complaint, the cases with a longer febrile period and with free contagion in the tube had a significantly lower number of infected cells in the blood than were detected in cases with a shorter febrile period and without free contagion, an observation suggesting that the inflexibility of exanthem subitum is reflected by the degree of addition and dispersion of HHV-6 in the blood [43]. However, a high viral load has been linked to a greater severity of infection in cases of herpesvirus infection [44]. This is the first study that reports herpesvirus viral loads in RD patients. In our study, the mean viral loads observed for EBV and HCMV were greater than 7.0x10^4^ and 3.0x10^4^ copies/mL, respectively, and for HHV-6, they were greater than 6.0x10^5^ copies/mL. As already described in a study that compared the presence of active infection with the viral load of HHV-6, viral load values with an average of 10^5^ copies/mL are indicative of active infection for HHV-6 [45]. The viral loads observed in the present study were considered high compared to tissue samples, which showed loads between 10^2^−10^3^ copies/μg for HHV-6 and EBV [46, 47], and compared to newborn serum with 10^3^−10^4^ copies/μL of HCMV [48]. Human herpesvirus 6 had the highest detection frequency among the three viruses evaluated; however, it was not possible to make a comparison with other studies in Brazil and in the world, as most of the studies that analyzed RD and AFI only performed serological detection. In 2008, Vianna and collaborators found a seroprevalence of HHV-6 of 43.5% out of a total of 223 samples in the city of Niterói-RJ, with fever (93.8%) and disseminated exanthema (90.7%) in most cases [49].

In our study, HHV-7 was not detected. However, a study conducted in 2011 using the nested PCR technique detected HHV-7 in 6.4% (9/141) of RD cases in children (<4 years) [4]. The differences found in the prevalence of HHV-6 and HHV-7 may be related to the larger sample number used in the 2008 and 2011 studies. Another relevant point was the inclusion criteria of the studied populations, as both studies excluded samples previously positive for measles, rubella, dengue and B19V in the serological diagnosis, while our study included all RD and AFI patients without a previous etiological diagnosis.

There are no similar studies with this seroprevalence data for B19V and herpesviruses. This is not only an epidemiological problem but also a public health problem, because these infections could become clinically significant in some populations.

In Brazil and in other Latin American countries, the current triple epidemic of arboviruses, including dengue, Zika and chikungunya, increased the challenge for clinicians to diagnose AFI and RD on a syndrome-based approach. The concurrent circulation of these viruses highlights a need to conduct differential laboratory diagnoses for AFI and RD, since there is no clinical algorithm robust enough to distinguish between the viruses that share remarkably similar clinical manifestations as Parvovirus B19 and herpesviruses. Rash was observed in all patients below 18 years of age (children and adolescents) included in the study. The most prevalent signs and symptoms among infected patients were fever (81.8%), pruritus (36.4%), diarrhea (27.3%), headache, nausea, and myalgia (18.2%).

The rash in EBV-positive patients was disseminated or on the lower limbs, progressing to spreading throughout the body. Infectious EBV mononucleosis with rash and fever, although more common in adolescents and young adults, can also be seen in children [50]. HCMV-positive patients had fever (100%), headache, pruritus, nausea (66.6%) and vomiting, myalgia, and diarrhea (33.3%). The exanthemas appeared in the regions of the trunk and head, progressing in a disseminated way throughout the body.

The profile of symptoms associated with HHV-6 infection found in the current study included fever (100%), pruritus and diarrhea (33.3%), and myalgia and vomiting (16.6%), corroborating previous findings in the literature [49]. Most rash (66.7%) in HHV-6 infections started in the head or neck and progressed to the whole body in a diffuse manner, confirming the description found by Korman and collaborators [51].

All RD-B19V-positive patients (3/3) had anemia, headache, and a local rash on the head. In relation to the AFI-B19V-positive patients, all but one (3/4) had anemia. These clinical manifestations are commonly found in Parvovirus B19 infection [52]. Although all patients with anemia progress to a good outcome, this clinical finding deserves attention, as it is an important aggravation of the infection and can lead to worse outcomes, mainly in immunocompromised patients [53, 54].

Even with the presence of these clinical signs and symptoms, no finding is pathognomonic of RD and AFI, which reinforces the importance of laboratory diagnosis.

The detection of B19V, EBV, HCMV, HHV-6 and 7 was possible using qPCR, demonstrating the importance of molecular diagnosis in identifying the etiologic agent in cases of RD and AFI with undefined etiology. The prevalence of these viruses highlights the importance of differential diagnoses for these infections in the face of those diseases, not only for epidemiological purposes but also for the proper management of the patient.

## Supporting information

S1 Table Prototype sequences used in phylogenetic analysis of the present study. (DOCX)
