# Supplementary material for: Beyond arboviruses: A multicenter study to evaluate differential diagnosis of rash diseases and acute febrile illness cases in Rio de Janeiro, Brazil
Source: PLoS One. 2022 Jul 29;17(7):e0271758. doi: 10.1371/journal.pone.0271758 (PMC9337664; doi:10.1371/journal.pone.0271758)
Supplement: S1 Table — (DOCX) [file pone.0271758.s001.docx]

| **Genbank**  **number** | **Genotype** | **Strain** | **Origin** | **Reference** |
| --- | --- | --- | --- | --- |
| M13178 | 1a | B19-Au | USA | Shade et al., 1986 |
| M24682 | 1a | B19-Wi | United Kingdom | Blundel et al., 1987 |
| U38546 | 1a | BrIII | RJ/Brazil | Cruz et al., 1988 |
| U38508 | 1a | IRE1 | Ireland | Erdman et al., 1996 |
| Z68146 | 1a | Stu | Germany | Hicks et al., 1996 |
| AF162273 | 1a | HV | Finland | Hokynar et al., 2000 |
| DQ293995 | 1a | C39 | Belgium | Parsyan et al., 2006 |
| EF089179 | 1a | PA79055BR | PA/Brazil | Freitas et al., 2007 |
| EF089209 | 1a | PA91018BR | PA/Brazil | Freitas et al., 2007 |
| EU478562 | 1a | S2337GerB05 | Germany | Norja et al., 2008 |
| EU478578 | 1a | S2827GerB04 | Germany | Norja et al., 2008 |
| JN211168 | 1a | 176937 | Netherland | De Backer et al., 2012 |
| KC013321 | 1a | 09BRSP6896 | SP/Brazil | Da Costa et al., 2013 |
| KP115293 | 1a | RJ011 | RJ/Brazil | Pereira et al., 2014 |
| KP115298 | 1a | RJ667 | RJ/Brazil | Pereira RFA, 2014 |
| KP115303 | 1a | RJ676 | RJ/Brazil | Pereira RFA, 2014 |
| KP115304 | 1a | RJ677 | RJ/Brazil | Pereira RFA, 2014 |
| KP115308 | 1a | RJ695 | RJ/Brazil | Pereira RFA, 2014 |
| KP115309 | 1a | RJ696 | RJ/Brazil | Pereira RFA, 2014 |
| KP115310 | 1a | RJ730 | RJ/Brazil | Pereira RFA, 2014 |
| KP115312 | 1a | RJ772 | RJ/Brazil | Pereira RFA, 2014 |
| KP115316 | 1a | RJ816 | RJ/Brazil | Pereira RFA, 2014 |
| KP115318 | 1a | RJ903 | RJ/Brazil | Pereira RFA, 2014 |
| KP115319 | 1a | RJ1409 | RJ/Brazil | Pereira RFA, 2014 |
| KP115321 | 1a | RJ1445 | RJ/Brazil | Pereira RFA, 2014 |
| KP115322 | 1a | RJ1452 | RJ/Brazil | Pereira RFA, 2014 |
| KP115323 | 1a | RJ1515 | RJ/Brazil | Pereira RFA, 2014 |
| KP115324 | 1a | RJ1524 | RJ/Brazil | Pereira RFA, 2014 |
| DQ357064 | 1b | Vn147 | Vietnam | Toan et al., 2006 |
| DQ357065 | 1b | Vn115 | Vietnam | Toan et al., 2006 |
| AY044266 | 2 | LaLi | Finland | Hokynar et al., 2002 |
| AY064475 | 2 | A6 | Italy | Nguyen et al., 2002 |
| AY064776 | 2 | A6 | Italy | Nguyen et al., 2002 |
| AJ717293 | 2 | Berlin | Germany | Liefeldt et al., 2004 |
| AY903437 | 2 | IM-81 | Germany | Blumel et al., 2005 |
| DQ333426 | 2 | BN31.2 | Germany | Schneider et al., 2008 |
| AJ249437 | 3a | V9 | France | Nguyen et al., 1999 |
| AY582125 | 3a | Gh3051 | Ghana | Parsyan et al., 2007 |
| DQ234775 | 3a | D1599 | Ghana | Parsyan et al., 2007 |
| DQ234769 | 3a | R0416 | Ghana | Parsyan et al., 2007 |
| DQ234771 | 3a | R0277 | Ghana | Parsyan et al., 2007 |
| AY083234 | 3b | D91.1 | France | Servant et al., 2002 |
| DQ408304 | 3b | BN60.3 | Germany | Schneider et al., 2008 |
| DQ408303 | 3b | BN59.3 | Germany | Schneider et al., 2008 |
| DQ408305 | 3b | BN30.3 | Germany | Schneider et al., 2008 |
| DQ408302 | 3b | BN58.3 | Germany | Schneider et al., 2008 |
| DQ234778 | 3b | R0693 | Ghana | Parsyan et al., 2007 |
| DQ234779 | 3b | R0748 | Ghana | Parsyan et al., 2007 |
| AY582124 | 3b | Gh2768 | Ghana | Parsyan et al., 2007 |

Supplementary table. Prototype sequences used in phylogenetic analysis of the present study
